# Mock Juror Perceptions of a Young Defendant With Developmental Language Disorder: A Mixed‐Methods Study

**DOI:** 10.1111/1460-6984.70060

**Published:** 2025-05-30

**Authors:** Jasmine Horsham, Katie Maras, Danaë Stanton Fraser, Ellie Barker, Hannah M. Hobson, Michelle C. St Clair

**Affiliations:** ^1^ Department of Psychology University of Bath Bath UK; ^2^ Department of Psychology University of York York UK

**Keywords:** content analysis, credibility perceptions, developmental language disorder, jurors, mock jury, neurodivergence, young defendant

## Abstract

**Introduction:**

Developmental language disorder (DLD), difficulties with using and/or understanding language, is highly prevalent in young offenders but is often undiagnosed. Even if there is a DLD diagnosis, it may not be deemed relevant to disclose to jurors. This study aimed to investigate whether the provision of a diagnostic label and information about DLD influenced mock juror perceptions of a young defendant.

**Method:**

Following the method of Maras et al. (2019), 158 participants read a fictional case study of a young defendant who was in court after assaulting a police officer in a misunderstanding at a train station. Half (*n* = 79) of the participants were informed that the defendant had DLD, and half (*n* = 79) were uninformed. Participants rated the defendant on his credibility (cognitive functioning, honesty and likeability) and culpability (blameworthiness, guilty verdict and sentencing leniency). They also indicated whether they thought the defendant committed the crime because of the situation he was in, because he was a bad person or both. Participants were asked to explain their reasoning behind each rating.

**Results:**

Participants in the informed condition viewed the defendant as significantly more credible and less culpable and were less likely to assign him a guilty verdict. Content analysis revealed four themes: anger, mitigating factors, communication, and situational context and police officers. Participants in the informed condition were more empathetic towards the defendant on all themes.

**Conclusion:**

Findings highlight the need for better detection of DLD in young people standing trial alongside the value of providing jurors with a defendant's diagnostic information.

**WHAT THIS PAPER ADDS:**

*What is already known on the subject*

- It is well established that youth and young adult offenders have a high rate of undiagnosed developmental language disorder (DLD). However, very little is known about how juries may perceive defendants with DLD. One study of a nonviolent offence found that defendants with a disclosed diagnosis of DLD were viewed as more likeable and honest and less blameworthy and cognitively able, but there was no difference in guilty verdicts or sentencing dependent on DLD diagnosis disclosure.

*What this paper adds to the existing knowledge*

- This study looks at mock jury perceptions of a defendant with DLD who is charged with a violent offence. We found that when mock jurors were informed of the defendant's DLD diagnosis, they rated the defendant not only as more credible (more likeable and honest) but also less culpable, with a reduction in guilty verdicts and reduced sentencing when the defendant was found guilty.

*What are the potential or actual clinical implications of this work?*

- This study indicates that it may be beneficial for defendants with DLD to have their diagnosis disclosed at trial. The results indicate this could lead to a better, more nuanced understanding of the defendant and the motivations and drivers behind their actions. Increased provision of SLT assessment and diagnosis of offenders might be useful in order to facilitate this disclosure at trial.

## 1 Background

Language is a vital aspect of communication involving the understanding and use of words to convey information (Bishop et al. 2016). Approximately 7.6% of UK children have developmental language disorder (DLD; Norbury et al. 2016), which involves persistent difficulties with using (expressive) and/or understanding (receptive) language (Bishop et al. 2017). However, DLD is critically under researched. McGregor (2020) demonstrated that, between 2010 and 2019, there were 27 and 14 times more research publications into autism spectrum disorder (ASD) and attention deficit/hyperactivity disorder (ADHD), respectively, than into DLD. This is despite DLD having a greater prevalence than both conditions and a greater impact on life outcomes, as rated by clinical experts, than ADHD (Bishop 2010).

Language difficulties, such as those experienced by individuals with DLD, are particularly common in young offenders. Indeed, language impairment may be a risk factor for youth offending (e.g., Brownlie et al. 2004). A systematic review by Anderson et al. (2016) reported language impairments are associated with higher rates of youth offending. Similarly, another meta‐analysis also found the language skills of young offenders to be substantially impaired compared to nonoffending peers. Studies in the UK also found below‐average language abilities in young offenders (e.g., Bryan et al. 2007). There appear to be no significant differences in language impairment rates between male and female young offenders (e.g., Chow et al. 2022).

DLD in particular is disproportionately overrepresented in youth offending populations, with language difficulties reaching the clinical threshold for DLD affecting up to 60% of young offenders (e.g., Snow and Powell 2008; Winstanley et al. 2021). Furthermore, within a sample of 145 young offenders those with DLD were 2.6 times more likely to reoffend within 1 year of their court order than young offenders without DLD (Winstanley et al. 2021).

According to the Royal College of Speech and Language Therapists (RCSLT), all young offenders in England are screened on their language ability, using the assessment tool AssetPlus (RCSLT 2023). AssetPlus is used by approximately 40% of practitioners to write presentence reports, which are concise documents provided to the court when a defendant is younger than 18 (Picken et al. 2019). However, screening of speech, language and communication needs specifically, which DLD falls under, is only reported in the AssetPlus module relating to the tailoring of interventions. Consideration of speech, language and communication needs is not mentioned in the UK Government's (2022) guidance for inclusion within presentence reports. This is a critical limitation considering the prevalence of DLD in youth offending populations, especially since language impairments identified in many studies were undiagnosed before study participation. For example, of the 60% of 145 young offenders identified to have DLD in Winstanley et al.’s (2021) study, none were diagnosed before the start of the study, and only two had previously participated in a Speech and Language Therapist‐led intervention (Winstanley et al. 2019). Furthermore, Bryan et al. (2015) found that whilst 30% of 118 males in a secure children's home scored at least 1.5 standard deviations below the mean on language tests (indicative of DLD), only two had previously identified speech and language difficulties.

It may be that only unidentified DLD is associated with offending. Supporting this, Mouridsen and Hauschild (2009) found no significant difference in conviction rates between adults without DLD and adults with a childhood diagnosis of DLD. Winstanley et al. (2018) found that adults with identified DLD were less likely to have contact with police compared to age‐matched peers. This demonstrates the need for early identification of and intervention for DLD to reduce engagement with Youth Justice Services.

As discussed above, DLD is highly prevalent in youth offending populations. Due to the aforementioned potential missed diagnoses and a lack of public awareness of the disorder, however, jurors on these cases may not be aware when a defendant has DLD. A study of 11 lawyers in America by LaVigne and Van Rybroek (2014) found that none were aware of language disorders being a diagnosable condition, and none recalled seeing language disorders mentioned in clients’ records. This was despite the lawyers acknowledging that many of their clients seemed to struggle with language. A survey by Thordardottir et al. (2021) with 1519 general population respondents across Europe found, on average, 60% were aware of Childhood Language Impairment (now termed DLD); however, this public awareness ranged considerably between different European countries, from 13% to 93%. A study of public awareness of DLD in Australia found that only 19.9% of 272 respondents were aware of DLD, compared to 97.4% for ASD (Kim et al. 2023), suggesting a striking lack of awareness compared to other disorders. In other European countries, public awareness of DLD also appears to be lacking. Kuvač Kraljević et al. (2022) found that although around 70% of 287 participants were aware of DLD, only 5%–42% across three countries (Croatia, Italy and Slovenia) were able to provide a sufficient definition of the disorder.

Additionally, recent research has extended this research with the general public to show a similar lack of awareness of DLD within law enforcement professionals. Benes and Lund (2024) showed that 69% of a law enforcement sample in Texas had not heard of DLD and of those that had, only 28% were able to give an accurate definition. More positively, on a credibility subscale measuring whether communication difficulties related to reduced credibility (e.g., relating communication difficulties to lying) in offenders, the sample was less likely to attribute blame or culpability based on communication characteristics, such as difficulty communicating and nonresponses. However, there was substantial variability on this measure, indicating some professionals would attribute blame based on characteristics related to communication difficulties. The sample was overwhelmingly in favour of making accommodation for communication difficulties, with 91% of the sample endorsing more training on DLD and communication difficulties. Considering both the low public awareness and the lack of awareness in law enforcement, jurors may not be informed that a defendant has DLD due to missed diagnoses. Furthermore, they will likely not suspect this diagnosis if they are not aware of the disorder and its characteristics. Therefore, it is important to investigate whether informing jurors that a defendant has DLD and providing a definition of DLD impacts their judgements. In England and Wales, where the judge deems that it will assist the jury, they may provide them with written materials. This may include expert evidence summaries; for example, scientific evidence detailing the effect of a defendant's neurodevelopmental diagnosis.

Furthermore, recent experimental research has shown that suspects with DLD are judged more harshly than suspects without DLD. In a study by Spaulding and Blewitt (2024), an interrogation was recorded with suspects who either had DLD or did not have DLD. Half of the sample had actually committed the mock crime that the interrogation focused on. The results indicated that mock jurors were poor at identifying guilty suspects, regardless of DLD status, but that individuals with DLD were 2.68 times more likely to be judged as guilty than individuals without DLD. This increased to 2.80 times when the individual was, in fact, guilty of the offence. Around 68% of the ‘not guilty’ individuals with DLD were rated as guilty, compared to only 45% of the ‘not guilty’ individuals without DLD. This indicates that behaviours and mannerisms linked to DLD may increase the likelihood of conviction, even when they did not commit any offence. Critically, however, this study did not look how disclosing a diagnosis may change perceptions.

Providing jurors with diagnostic information may influence their perceptions of a defendant when considering Kelley's (1987) discounting principle. The principle suggests that the seeming cause of a behaviour may be dismissed when other potential causes are available. This principle has been applied to the context of juror perceptions in other neurodevelopmental disorders. For example, Blackhurst et al. (2022) contend that the provision of an ASD label provides jurors with an alternative and more acceptable cause of criminal and courtroom behaviour exhibited by a defendant. When reevaluating a defendant after being informed that he had ASD, participants in this study were more empathetic and deemed the defendant to be more honest and less blameworthy, particularly if they had more knowledge about psychological conditions, such as ASD. Further support for applying this principle to legal settings was found by Berryessa and colleagues (2015); participants in this study tended to view an autistic defendant's behaviour to be out of his control due to the ASD diagnosis.

Maras et al. (2019) investigated how UK mock jurors’ perceptions of a defendant differed when provided with background information detailing that the defendant had ASD. Participants rated the defendant's credibility (cognitive functioning, honesty and likeability) and culpability (blameworthiness, guilty verdict and sentencing leniency) and provided qualitative descriptions explaining their ratings. Participants who were informed that the defendant had ASD viewed him to be more honest and likeable, and less blameworthy, compared to those who were not informed of the defendant's diagnosis. The informed condition was also less likely to suggest a guilty verdict, and those who deemed the defendant guilty suggested more lenient sentencing compared to the uninformed condition. Most viewed the defendant as honest, although he was seen as more likeable by those in the ASD label condition due to a perception that autistic people cannot lie. This last finding raises an interesting point in relation to the discounting principle, in that not all jurors will have an accurate understanding of neurodevelopmental disorders and mental health conditions. Perceptions may be altered in a too lenient direction (e.g., autistic defendants cannot lie) or in a more negative, punitive direction, perhaps based on inaccurate prejudices and stereotypes (e.g., schizophrenic individuals are all violent).

Using a similar method, Hobson et al. (2023) looked at whether similar effects were found with a DLD diagnosis in a scenario involving 22‐year‐old defendant accused of a nonviolent crime. In this study of 143 mock juror participants, those who were informed that the defendant had DLD viewed him as more likeable, more honest and less blameworthy, but also less cognitively able. They did not find a significant difference between groups in the likelihood of finding the defendant guilty, or in judgements of sentencing leniency. Their content analysis of participants’ explanations for their ratings found that those in the informed condition were more likely to refer to the defendant's level of cognitive functioning when justifying sentencing leniency, likeability and blameworthiness. However, there were some key differences between this study and Maras et al. (2019). Primarily, Hobson et al.’s (2023) study involved a defendant committing a nonviolent crime, whereas Maras et al. (2019) described a violent crime. Given that a violent crime is the most common offence committed by young offenders with DLD (Winstanley et al. 2021), it is important to assess if Hobson et al.’s findings are replicated when the crime is of a violent nature. Additionally, most research finding an association between DLD and offending focuses on youth offending, whereas the defendant in Hobson et al.’s (2023) study was 22 years old.

## 2 Aims and Objectives

The current study adapted Maras et al.’s (2019) study of a defendant with ASD to assess whether provision of a diagnosis—label and background information about the condition—affects how mock jurors perceive a defendant with DLD. In this case, the defendant in the case study was a young male committing a violent crime. A mixed‐methods design was adopted, in line with Maras et al.’s (2019) study, and to allow a more comprehensive understanding of this scarcely studied topic area. We predicted that jurors who were informed that the defendant had DLD would perceive him to be more likeable, more honest and less blameworthy. We also measured DLD awareness in both conditions in order to explore how awareness of DLD, and suspicion of a developmental disability or mental health condition in the uninformed condition, influences perceptions of the defendant.

## 3 Materials and Methods

### 3.1 Participants

Participants (*N* = 158 after exclusions; *N* = 172 before exclusions) were recruited via Prolific (www.prolific.co), an online platform facilitating paid participation in research studies. Participants received £10.59/h for their participation, which averaged around £1.70 per participant (between 9 and 10 min). Half of the participants (*N* = 79) were randomly assigned to the informed condition and half (*N* = 79) were assigned to the uninformed condition. There were no significant differences between the two groups on gender, employment status or whether they had studied psychology. However, the informed condition was slightly younger than the uninformed condition (*t*(156) = −2.09, *p* < 0.05, *d* = −0.33). The assignment sequence was determined by the survey platform, Qualtrics (www.qualtrics.com). Participant demographics are reported in Table 1.

**TABLE 1:** Demographic information of participants.

Characteristic		Informed	Uninformed	Total
		(*n* = 79)	(*n* = 79)	(*n* = 158)
Gender	Male *n* (%)	15 (18.99%)	22 (27.85%)	37 (23.42%)
	Female *n* (%)	64 (81.01%)	56 (70.89%)	120 (75.95%)
	Nonbinary *n* (%)	0	1 (1.27%)	1 (0.63%)
Mean age (SD)		38.87 (*13.90*)	43.58 (*14.39*)	41.23 (*14.30*)
Employed	Nonstudents *n* (%)	47 (59.49%)	55 (69.62%)	102 (64.56%)
	Students *n* (%)	2 (2.53%)	1 (1.27%)	3 (1.90%)
Unemployed	Nonstudents *n* (%)	26 (32.91%)	21 (26.58%)	47 (29.75%)
	Students *n* (%)	4 (5.06%)	2 (2.53%)	6 (3.80%)
Studied psychology or psychiatry to degree level or higher *n* (%)		6 (7.59%)	3 (3.80%)	9 (5.70%)

Participants were eligible for UK jury duty; those who were ineligible (*N* = 10) or who did not complete final consent (*N* = 4) were excluded. To be eligible for UK jury duty, participants had to be aged between 18 and 75 and have lived in the UK, Channel Islands or the Isle of Man for a period of at least 5 years since the age of 13. Additionally, they must not have been sentenced to imprisonment, or a detention of at least 5 years, and must not currently be on bail in criminal proceedings. Participants also must not have lacked capacity by way of the Mental Capacity Act (2005); specifically, they must not have a condition that affects their decision‐making, such as brain damage. A small number of participants (*N* = 2) did not complete all qualitative explanations for their ratings, but provided quantitative ratings and so were not excluded from the study. Ethical approval was obtained from the ethics board of the (PREC code UG 22‐014).

### 3.2 Materials

The informed condition was told that the defendant, Mr Edwards, has DLD and was provided with a description of what DLD is and how it affects the defendant. The uninformed condition was not told that the defendant has DLD. This format followed that of Maras et al.’s (2019) study. Participants were all provided with a vignette detailing the defendant's case and court appearance. These excerpts were read, and corrections for accuracy were suggested by three Speech and Language Therapists who work with young offenders. All participants completed an online credibility and culpability questionnaire via Qualtrics. This was comprised of multiple 7‐point Likert scales as used by Maras et al. (2019), although the defendant's name was changed from ‘Mr Parsons’ to ‘Mr Edwards’. We additionally included a question on DLD awareness at the beginning of the questionnaire for the informed condition and at the end of the questionnaire for the uninformed condition, asking if the participants thought Mr Edwards had ‘any developmental disability or mental health condition’.

Participants rated the defendant's credibility across three questions, pertaining to his level of cognitive functioning, honesty and likeability on 7‐point Likert scales (e.g., 1 = ‘not at all likeable’ to 7 = ‘very likeable’). Perceptions of the defendant's culpability were explored in relation to his blameworthiness (Likert scale: 1 = ‘not at all to blame’ to 7 = ‘very blameworthy’) and whether he should receive a ‘guilty’ or ‘not guilty’ verdict. Participants were asked to rate their confidence in this judgement of guilt (1 = ‘not at all confident’ to 7 = ‘very confident’). If a guilty response was given, a follow‐up question asked how harshly he should be sentenced (1 = ‘very leniently’ to 7 = ‘very harshly’). Finally, participants were also asked to indicate why they believed the defendant committed the crime, either because of the situation he was in, because he was a bad person or both. After giving each rating, participants responded to an open‐ended question asking them to provide an explanation of why they had given this rating. Full study materials can be found on the Open Science Framework (https://osf.io/z5trk/).

### 3.3 Procedure

This study was an online study conducted within the Prolific recruitment platform. After all participants read the information sheet and completed initial consent and eligibility checking, participants in the informed condition indicated whether they had heard of DLD before reading the description of Mr Edwards’ DLD. Participants in both conditions then read excerpts titled ‘Case summary’ and ‘At court’. The ‘case summary’ described an incident at a train station wherein Mr Edwards had a misunderstanding with a ticket officer and became angry, causing the police to be called. When questioned by the police, Mr Edwards became increasingly agitated and hit the police officer. The ‘at court’ excerpt displayed some of the conversation between Mr Edwards and a lawyer. The actions and behaviour of Mr Edwards aligned with current literature on DLD, such as impaired sentence comprehension (Montgomery and Evans 2009) whilst talking with the lawyer, and aggression (Özcebe et al. 2020) towards the police officer. After reading these excerpts, all participants completed the credibility and culpability questionnaire. Participants in the uninformed condition were then asked if they thought the defendant had any developmental disability or mental health condition and if they had heard of DLD. Finally, all participants gave final consent and were debriefed on the nature of the study.

### 3.4 Quantitative Analysis

For the credibility questions, we conducted a multivariate analysis of variance (MANOVA) with the combined ratings, which was followed by univariate analyses of variance (ANOVAs) if the main effect of diagnosis disclosure was significant. As the culpability questions could not be combined in a similar manner as the credibility questions (due to different scales of measurement and differences in constructs), a *t* test was conducted to evaluate the level of blameworthiness, confidence in guilty judgement and leniency (when the defendant was judged guilty) between the two disclosure conditions. A chi square was conducted to evaluate whether the binary guilty verdict and awareness of DLD differed between conditions. A Fisher–Freeman–Halton exact test, which is suitable when a continency table is larger than 2 × 2, was used to evaluate whether the attribution of blame to the situation, the defendant or both differed between the conditions (Freeman and Halton, 1951).

### 3.5 Qualitative Analysis

A content analysis was conducted by the first author to analyse participants’ explanations for each rating. All responses were analysed together, rather than question‐by‐question. Codes were derived from the data, and categories and themes were formed from these codes. Intercoder reliability was assessed by comparing the codes between the first author and a coresearcher on 25% of the data (40 participant responses). A score of 1 was marked for each agreement on the presence or nonpresence of a code. Intercoder reliability was 0.91. This *κ* score exceeds 0.81 and so indicates almost perfect agreement according to Cohen (1960; McHugh 2012). Chi square tests were then run to determine significant differences in the frequency of responses within themes and categories between the informed and uninformed conditions (Vaismoradi et al. 2013). Content analysis was chosen to allow evaluation of the frequencies across categories of people, to facilitate a more complete understanding the effect of diagnosis disclosure on the reasoning underlying the culpability and credibility assessments. Please see the Appendix for the codes and further quotes.

### 3.6 Accessing Materials

The study materials are available and can be found on the Open Science Framework (https://osf.io/z5trk/). Analysis code is available on the Open Science Framework (https://osf.io/z5trk/). The study data is not available for ethical reasons, as permission was not obtained from participants for their data to be made open access.

## 4 Results

### 4.1 Quantitative Ratings

All assumptions for the MANOVA, *t* tests, chi square and Fisher–Freeman–Halton tests were met.

#### 4.1.1 Credibility

A one‐way MANOVA indicated a statistically significant difference with a large effect size between the informed and uninformed conditions on the combined ratings of credibility, *F*(3,154) = 9.42, *p* < 0.001, Wilks’ Λ = 0.85, partial *η* ^2^ = 0.16. Univariate tests revealed that cognitive functioning was rated significantly lower with a medium effect size by participants who were informed of the defendant's DLD than those who were not, *F*(1,156) = 10.08, *p* = 0.002, partial *η* ^2^ = 0.06. Participants who received DLD information also rated the defendant as significantly more honest, *F*(1,156) = 5.16, *p* = 0.03, partial *η* ^2^ = 0.03 and likeable, *F*(1,156) = 13.94, *p* < 0.001, partial *η* ^2^ = 0.08, than those who received no information about his diagnosis (Table 2). The finding on honesty was a small effect size, while the effect in likability was a medium effect size.

**TABLE 2:** Participant ratings of the defendant's credibility and culpability.

			Mean (SD)
Credibility	Cognitive functioning	Informed	2.97 (*1.15*)
		Uninformed	3.61 (*1.34*)
	Honesty	Informed	5.85 (*1.12*)
		Uninformed	5.42 (*1.26*)
	Likeability	Informed	3.71 (*1.33*)
		Uninformed	2.97 (*1.13*)
Culpability	Blameworthiness	Informed	3.86 (*1.57*)
		Uninformed	5.25 (*1.29*)
	Confidence in guilt	Informed	4.95 (*1.36*)
		Uninformed	5.29 (*1.48*)
	Sentencing leniency	Informed	3.08 (*1.45*)
		Uninformed	2.39 (*1.13*)

#### 4.1.2 Culpability

**FIGURE 1:**
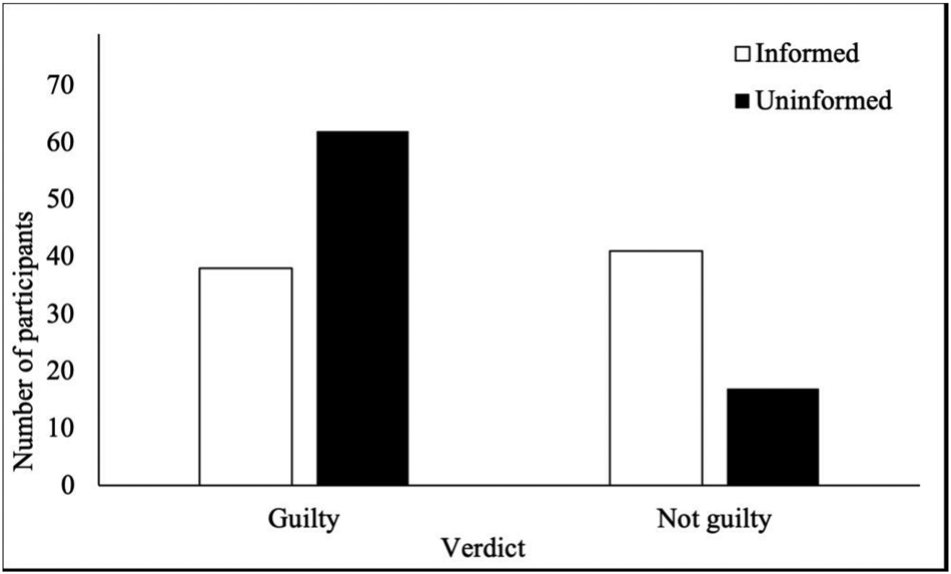
Number of participants assigning the defendant a guilty or not guilty verdict, by condition.

An independent *t* test indicated that participants who were not informed about the defendant's DLD deemed him to be significantly more blameworthy, with a large effect size, than participants who were informed that he had DLD, *t*(156) = 6.11, *p* < 0.001, *d* = 1.43. Further, a chi square test determined a significant association between condition and assigning the defendant a guilty verdict, *χ* ^2^(1) = 15.69, *p* < 0.001, *φ* = −0.32, indicating a medium effect size. Participants who were not informed of the defendant's DLD diagnosis were more likely to assign a guilty verdict than those who knew about his DLD (78.48% vs. 48.10%), see Figure 1.

There was no significant difference in participants’ confidence in their judgement of guilt between the informed condition and the uninformed condition, *t*(156) = −1.5, *p* = 0.13, *d* = 1.42, although the effect size was large. However, for those who deemed the defendant guilty, participants who were unaware of his DLD diagnosis gave significantly harsher sentences (than those who were informed of his DLD, *t*(98) = 2.45, *p* = 0.02, *d* = 1.34, with a large effect size.

**FIGURE 2:**
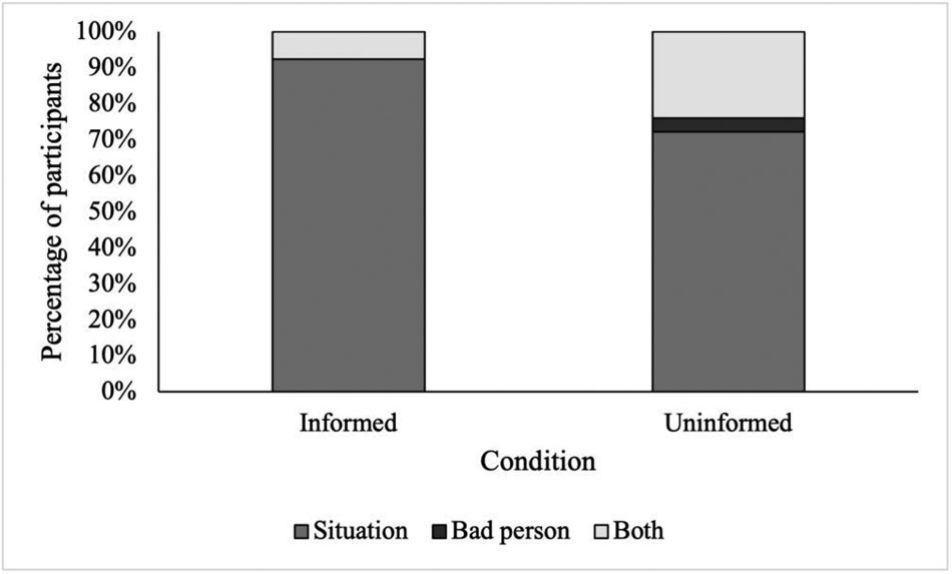
Percentage of participants deeming the defendant to have committed the crime due to the situation, being a bad person, or both, by condition.

The Fisher–Freeman–Halton exact test demonstrated that there was a statistically significant association between diagnosis information condition and deeming the defendant's actions to be a result of either the situation, because he is a bad person or both (*p* = 0.002). The effect size was moderate, Cramer's *V* = 0.27, *p* = 0.003. Overall, awareness of a DLD diagnosis led to fewer participants attributing blame on Mr Edwards; rather, they were more likely to attribute blame to the situation he found himself in, see Figure 2.

#### 4.1.3 DLD Awareness

Awareness of DLD was low, with 15.19% (*n* = 12) of the informed condition and 10.13% (*n* = 8) of the uninformed condition having previously heard of the disorder. A chi square test determined a nonsignificant association between DLD awareness and condition, *χ* ^2^(1) = 0.92, *p* = 0.34, *φ* = 0.08. None of the participants in the uninformed condition thought that the defendant may have DLD or a language impairment, although many (*n* = 21, 26.58%) suggested that he may have ASD. There was a significant difference in guilty verdicts between participants in the uninformed condition who thought the defendant may have ASD and those who did not. Participants were more likely to choose not guilty if they thought the defendant had ASD, *χ* ^2^(1) = 4.65, *p* = 0.031, *φ* = 0.24, indicating a small‐to‐medium effect size.

One participant suggested that the defendant may have a disorder affecting communication.

### 4.2 Qualitative Responses

Four higher order themes emerged from the data: anger, mitigating factors, communication and ticket and police officers could have done more. Anger was frequently discussed in terms of the defendant appearing aggressive or frustrated, thus aggression and frustration formed anger subthemes. The frequencies of the themes were compared across the informed and uninformed conditions using chi square tests (see Table 3).

**TABLE 3:** Summary of themes, example quotes and chi square test results on the content analysis of participants’ open‐ended responses.

Theme (category)	Example quote	Chi square result
Anger	‘I think he is very honest however, due to his disability he is coming across as aggressive but I do not believe he is aggressive at all but more to the fact that his frustration is coming across as aggression when this is not the case.’ (informed)	*χ* ^2^ *=* 6.29, *p* = 0.012^*^ Cramer's *V* = 0.20 Informed mentioned: 46.84% (*n* = 37) Uninformed mentioned: 66.66% (*n* = 52)
Anger: frustration	‘He became frustrated with being unable to be understood or to understand.’ (informed)	*χ* ^2^ *=* 4.31, *p* = 0.038^*^ Cramer's *V* = 0.22 Informed mentioned: 56.76% (*n* = 21) Uninformed mentioned: 34.62% (*n* = 18)
Anger: aggression	‘He was aggressive, uncommunicative and unpredictable.’ (uninformed)	*χ* ^2^ *=* 3.66, *p* = 0.056 Informed mentioned: 29.73% (*n* = 11) Uninformed mentioned: 50.00% (*n* = 37)
Mitigating factors	‘Although he had reason to become annoyed he should not have hit anyone. However I am not fully convinced he does not have a condition that could have caused him to respond the way he did i.e. Could he have autism.’ (uninformed)	*χ* ^2^ = 0.68, *p* = 0.41 Informed mentioned: 18.99% (*n* = 15) Uninformed mentioned: 14.10%
Communication	‘Mr Edwards clearly struggles to fully understand questions and scenarios put to him, and then struggles to provide explanations.’ (informed)	*χ* ^2^ *=* 6.35, *p* = 0.012^*^ Cramer's *V* = 0.20 Informed mentioned: 51.90% (*n* = 41) Uninformed mentioned: 32.05% (*n* = 25)
Ticket and police officers could have done more	‘Mr Edwards did react, however there were point where this could of be deescalated. The ticket officer, the policemen all have parts to play in “blame”.’ (uninformed)	*χ* ^2^ *=* 4.46, *p* = 0.035^*^ Cramer's *V* = 0.17 Informed mentioned: 22.78% (*n* = 18) Uninformed mentioned: 10.26% (*n* = 8)

* Significant at *p* < 0.05.

Participants regularly referred to the defendant's anger, as conveyed through his behaviour and speech. Of the 46.84% of participants in the informed condition who discussed the defendant's anger, over half (56.76%) described him as frustrated. Contrastingly, of the 66.66% of participants in the uninformed condition who mentioned anger, significantly fewer (34.62%) mentioned frustration. Participants in the uninformed condition were instead significantly more likely to describe the defendant as aggressive, with half discussing the defendant's aggression, compared to less than a third (29.73%) of participants in the informed condition. The overall anger and frustration findings had a small to medium effect size.

Participants in both conditions frequently discussed mitigating factors that could explain the defendant's actions, such as DLD in the informed condition and autism or mental health issues in the uninformed condition. Both conditions did not significantly differ in the frequency of mentioning mitigating factors, with 18.99% and 14.10% in the informed and uninformed conditions, respectively, discussing these.

Many participants also discussed that the defendant struggled with communication, particularly in terms of struggling to articulate himself and having difficulty understanding and responding to the questions the lawyer asked of him. Participants in the informed condition were more likely to discuss the defendant's communication difficulties than those in the uninformed condition. Over half (51.90%) of participants who were informed of the defendants’ DLD diagnosis discussed their communication, compared to less than one‐third (32.05%) of participants who were not told of their DLD diagnosis. This was a small to medium effect size (Table 3).

More participants (22.78%) who were informed of the defendant's DLD diagnosis also felt that the ticket and police officers could have done more to understand Mr Edwards and help him before the situation intensified, compared to 10.26% of the uninformed condition (a small to medium effect size). Chi square tests results on the qualitative data are shown in Table 3.

## 5 Discussion

This study adapted Maras et al.’s (2019) study of an autistic defendant to investigate whether mock jurors’ perceptions of a defendant were affected by the provision of a diagnosis of DLD alongside an explanation of the disorder. The informed condition viewed the defendant, Mr Edwards, as typically more credible (specifically more honest and likeable), although with lower cognitive functioning, compared to the uninformed condition. Those informed of the defendant's DLD also perceived him to be less culpable, in that he was deemed less blameworthy, and informed participants were more likely to suggest a not guilty verdict alongside more lenient sentencing if deemed guilty. Participants in the informed condition were also more likely to believe the situation caused the defendant to commit the crime, as opposed to him being a bad person or a combination of the two. Across our participant sample, DLD awareness ranged between 10.13% (uninformed) and 15.19% (informed), with none of the uninformed condition indicating that they thought the defendant may have a language disorder.

Participants’ explanations for their ratings formed four themes: anger, mitigating factors, communication and the role of the ticket and police officers. The defendant's anger was typically described as either aggression or frustration. Both conditions referenced these two categories, although participants in the informed condition discussed their frustration significantly more often than those in the uninformed condition. Participants in both conditions referenced mitigating factors, with this being DLD in the informed condition and mental health conditions or ASD in the uninformed condition. In terms of communication, significantly more of the informed condition discussed the defendant struggling with receptive and expressive aspects of communication, such as understanding questions and formulating responses. Lastly, participants discussed that the ticket and police officers could have done more to de‐escalate the situation, with significantly more participants in the informed condition mentioning this.

The quantitative findings in the current study largely aligned with those of Maras et al.’s (2019) study with an autistic defendant, indicating jurors’ more favourable perceptions when provided with diagnostic information about a defendant may not be disorder‐specific. However, some key differences in findings were identified between the present study and Hobson et al.’s (2023) study with a defendant with DLD. Although both studies found more favourable ratings of credibility and culpability when mock jurors were informed that the defendant had DLD compared to when they were not, Hobson et al. (2023) did not find a significant difference between these two conditions in suggesting a guilty or not guilty verdict. Although the present study involved a 17‐year‐old young defendant and Hobson et al. (2023) focused on a 22‐year‐old adult defendant, it is unlikely that the defendant's age could explain this difference. Warling and Peterson‐Badali (2003) found mock jurors did not differ in their likelihood of suggesting a guilty verdict when a defendant was aged 13, 17, or 25. However, it is possible that the nature of the crime could have impacted the difference between the current study and Hobson et al.’s (2023) study. Both the present study and Maras et al.’s (2019) study focused on a violent crime and found that the provision of diagnostic information was associated with less guilty verdicts. In contrast, Hobson et al.’s (2023) study featured a nonviolent crime involving theft. Filone et al. (2014) found that mock jurors perceived a nonviolent crime involving money more negatively than a violent crime, including through suggesting longer sentences. The authors suggested this finding might be related crime involving money (white collar crime) being a reflection of a greedy individual less likely to reform, whereas violent crimes may be due more due to the situation and unfortunate life circumstances. It is plausible that the nonviolent nature of the crime negated the effect of condition on juror ratings, hence the observed difference between Hobson et al.’s (2023) study and the current study.

Another key difference between the current study and Hobson et al.’s (2023) study that could explain the different findings is the content included within the excerpts given to participants. Hobson et al.’s (2023) background information referenced the defendant's history of antisocial behaviour, namely being accused of shoplifting alongside being excluded from school following behavioural issues. Neither the current study nor Maras et al.’s (2019) study of an autistic defendant mentioned prior disruptive behaviours of the respective defendants, instead only mentioning a tendency to be aggressive when anxious. It is plausible that participants in the informed condition in Hobson et al.’s (2023) study did not differ from those in the uninformed condition when assigning a guilty verdict or in sentencing leniency due to observing a potential pattern of antisocial behaviours in the defendant that is not observed in the current study nor in Maras et al.’s (2019) study. In the UK, the disclosure of previous convictions or misconduct without a criminal charge or conviction is not automatic and is most commonly allowed under bad character applications under the Criminal Justice Act 2003. These applications are made pretrial and are complex legal arguments decided by the trial judge. Further research investigating the effect of previous conviction disclosure on mock jury perception is needed to fully understand how these differences influence defendant perceptions.

Moreover, Hobson et al.’s (2023) study was more ambiguous as to whether the defendant had committed a crime. The defendant ran out of a store with a laptop, although it was left unclear as to whether he was attempting to steal the laptop or escape the situation. Contrastingly, in the current study, the defendant is stated to have hit a police officer, thus there is no ambiguity. This could explain the difference in culpability findings between the current study and Hobson et al.’s (2023) study.

Hobson et al.’s (2023) study did not mention the defendant's IQ but found that those informed of the defendant's diagnosis rated his cognitive functioning as lower. The cognitive functioning difference was replicated in the current study despite the defendant's IQ being described as normal in the vignette. Furthermore, differences in cognitive functioning were not found in Maras et al.’s (2019) study of an autistic defendant, indicating that people may associate DLD with lower cognitive functioning. This suggests that the presence of diagnostic information does not have a wholly positive impact on mock juror perceptions. Rather, participants may have assumed that individuals with DLD have poor cognitive functioning, indicating an incomplete understanding of the disorder. This study may have been the first occasion that participants were knowingly introduced to someone with DLD.

The present study was the first to report on the effect of diagnostic information on juror perceptions of the motivation for a defendant committing a crime. The association between provision of a diagnostic label and increased likelihood of believing the defendant committed the crime because of the situation he was in was supported by results of the content analysis, specifically the mitigating factors theme. Of the 18.99% of the informed condition who referred to the defendant's DLD, most did so in the context of explaining his presentation and behaviour, including suggesting that the defendant could not control his actions due to his disorder. These findings provide support for the application of Kelley's (1987) discounting principle to DLD‐specific criminal cases. In the case of this study, the potential of the defendant being a bad person as a cause of the crime may be discounted by the external presence of a diagnostic label and explanation, giving rise to considerations that the situation resulted in the defendant committing the crime. Importantly, some participants in the uninformed condition did suspect that the defendant could have a mental health condition or ASD, also considering that this could be a cause of his behaviour. Considering that the uninformed condition was, however, less likely to think that the situation caused the defendant to commit the crime, it may be that the mere presence of a behaviour associated with a disorder is insufficient to discount other causes. Rather, a confirmed diagnosis may be needed, supporting the importance of assessing defendants for language disorders so that jurors can be provided with this diagnostic information.

The findings also indicate that provision of a diagnostic label and information about DLD influences what aspect of a crime jurors tend to focus on. Those who were informed that the defendant had DLD often explained the defendant's actions as a sign of him being frustrated. Contrastingly, the uninformed condition typically referred to this behaviour as aggressive. The American Psychological Association defines frustration as the emotional state after being blocked from obtaining something that was expected, whereas aggression is considered to be a behaviour intending to cause harm (American Psychological Association 2023a, 2023b). Considering these definitions, the informed condition appears to have focused more so on the defendant's emotional state and so the reasoning behind his actions, whilst the uninformed condition tended to concentrate on the nature of his actions. This shift from behaviour to intentionality between the two conditions indicates that informing jurors of a defendant's DLD enables them to consider the reasoning behind his behaviours more so than when uninformed. This further highlights the importance of informing jurors if a defendant has DLD.

The content analysis also suggested that participants believed that the ticket and police officers were in part responsible for not deescalating the situation. This was more frequently discussed by the informed condition than the uninformed condition, with many believing the police officers should have recognised that the defendant had DLD. It is unclear if UK police officers are aware of DLD or language impairments in general due to a lack of research in this area, but recent research from Texas indicates a low rate of DLD awareness in law enforcement professionals (Benes and Lund 2024). A study of speech, language and communication needs awareness amongst police officers in Scotland found that 73.9% of Police Scotland officers had previously been concerned about a young person's receptive language (MacRae and Clark 2021). There is also limited information regarding whether UK police receive training on DLD or speech, language and communication needs in general. Metropolitan police recruits are mandated to complete a training package on neurodiversity and mental health, although it mainly focuses on mental health and not specific neurodiverse disorders (Metropolitan Police 2022). Therefore, participants’ expectations of police officers in the present study do not align with the reality of police officers’ abilities to deescalate situations when a young person has DLD; they are unlikely to have received training on this disorder. The current study suggests there may be public support for police officers to receive better training in conditions such as DLD and Benes and Lund's (2024) findings support a willingness of law enforcement professionals to engage in this training.

The current study had some limitations. First, most of our mock juror participants (75.95%) were female. Although research into gender differences suggests that females are more empathetic than males (Wuying et al., 2014), studies of mock jurors suggest otherwise. Guy and Edens (2003) found that females viewed a defendant described as psychopathic less favourably than males, for example, perceiving the defendant to be more dangerous. Moreover, Fischer (1997) found that female mock jurors were more likely to assign a defendant a guilty verdict than males. However, when these jurors deliberated in groups, there was only a significant difference in the verdict when the jury was nearly all or entirely female. Additionally, the studies by Guy and Edens (2003) and Fischer (1997) focused on criminal behaviour of a sexually violent nature. Females are statistically more at risk of being victims of sexual violence (Office for National Statistics 2021), which could have influenced the differential perceptions of the defendant between genders in these studies. Therefore, these findings may not provide sufficient support for potential gender differences affecting results in the current study. Future research investigating whether gender influences ratings of a defendant with DLD would provide a richer understanding of these issues.

A further limitation concerns suggestions amongst the uninformed condition of the defendant potentially having ASD. When asked if they thought the defendant had any developmental disability or mental health condition, a quarter of the uninformed condition suspected that the defendant may have ASD. Although DLD and ASD may share similar characteristics, as children with ASD may have a language disorder associated with autism (Mody and Belliveau 2013), DLD and autism cannot co‐occur according to current diagnostic understandings (Bishop et al. 2017). This finding suggests that some of the uninformed jurors’ perceptions of the defendants may have been influenced by the assumed presence of a disorder. Suspecting the defendant to have ASD did affect their ratings of the defendant, as mock jurors who thought the defendant might have ASD were likely to find the defendant guilty. However, this is still representative of a typical court case. It is probable that many jurors will hold judgements and make assumptions about a defendant, such as the presence of a disorder. As such, this is not a considerable limitation of the study.

### 5.1 Implications

The present study has important implications for legal proceedings. The findings demonstrate that the presence of behaviour related to a neurodevelopmental disorder without diagnosis leads to less favourable perceptions of a young defendant than when a diagnostic label and information are provided. Real‐life jurors should therefore be informed if a defendant has DLD in order to understand how their behaviour may be affected by their diagnosis. However, this is reliant upon DLD having been identified in the defendant, but there exist high rates of undiagnosed DLD in youth offending populations (Winstanley et al. 2021). As such, the current findings emphasise the necessity for presentence reports to include the results of screenings and assessments for speech, language and communication needs, rather than these only being considered when tailoring interventions. Future research could investigate whether juror perceptions of a defendant differ when a presentence report either includes or excludes results of speech, language and communication needs screening.

The importance of the current study is further emphasised through the finding of limited DLD awareness amongst jury‐eligible UK adults. Although awareness of DLD ranged from 10% (uninformed) to 15% (informed) between conditions, it is probable that an even lower number would have been able to provide a sufficient description of the disorder when considering Kuvač Kraljević et al.’s (2022) findings that only 40% of those aware of DLD have an accurate understanding of the disorder. This highlights the importance of the current study to advance our understanding of how mock jurors may perceive a defendant's disorder that they have likely not previously heard of. This finding exemplifies the need for an increased awareness of DLD in the UK. Campaigns have already been established with the intention of raising public awareness of DLD. For example, raising awareness of DLD provides information on DLD as well as resources that can be shared to raise awareness of the disorder. Although DLD awareness campaigns have yet to focus upon raising awareness specifically of the prevalence of DLD in young offenders. Although important, this should be done with caution by emphasising that DLD does not cause youth offending. Rather, a lack of diagnosis and intervention may increase one's likelihood of engaging in criminal behaviour.

## 6 Conclusion

This study builds on previous research suggesting that mock jurors perceive a defendant more favourably when provided with diagnostic information. Undiagnosed DLD is highly prevalent in youth offending populations, but jurors may not be aware when a defendant has DLD. The present study found that mock jurors perceived a young defendant as more credible and less culpable when informed of his DLD diagnosis. These findings have important implications, highlighting the need for better detection of DLD in young defendants as well as the benefits of providing jurors with diagnostic information. The results also call for a general increased awareness of the disorder, with a recommendation for DLD awareness campaigns to consider DLD in relation to youth offending. Future research should compare juror perceptions of a defendant when their presentence report does versus does not include speech language and communication needs screening results. This would support the present study's recommendation for the inclusion of these results within the presentence reports provided to courts.

## Ethics Statement

This research was approved by the University of Bath's Department of Psychology Research Ethics Committee (REF: UG 22‐014).

## Conflicts of Interest

The authors declare no conflicts of interest.

## Data Availability

The materials for this project are available on the Open Science Framework (https://osf.io/z5trk/). We did not collect permission from our participants to make their data open access, therefore the data is not available within a data archive.

## 1

The quotations are reproduced in verbatim as written by participants.

**Table:**

Theme	Category	Example quotes
Anger	Aggression	‘He is coming across aggressive, disconnected, refusing to make eye contact.’ (informed) ‘He is probably pitiable, but not likeable, given his tendency to aggression.’ (informed) ‘He was aggressive, uncommunicative and unpredictable.’ (uninformed) ‘The aggressive nature he displayed during the incident matched with his deminor in court.’ (uninformed) ‘He became aggressive and violent when faced with a situation he didn't like.’ (uninformed) ‘He obviously has tendencies to become aggressive and confrontational.’ (uninformed)
	Frustration	‘I think he is very honest however, due to his disability he is coming across as aggressive but I do not believe he is aggressive at all but more to the fact that his frustration is coming across as aggression when this is not the case.’ (informed) ‘He became frustrated with being unable to be understood or to understand.’ (informed) ‘In his own way he told the truth but because of his DLD it was misunderstood at station and he became frustrated at his own inability to communicate.’ (informed) ‘Mr Edwards clearly became frustrated by the incident and due to his cognitive functioning is unable to behave in the same way someone without DLD would.’ (informed) ‘He admitted his guilt and acted out of frustration rather than violence.’ (uninformed) ‘He did not do it out of malice, but just frustration at a miscommunication.’ (uninformed)
Mitigating factors	DLD diagnosis	‘His anger and frustration from the language disorder takes over.’ (informed) ‘The disorder takes over and wasn't meant to attack the police officer.’ (informed) ‘He isn't a bad person, as he is afflicted with a disorder that he can't control…or knows how to control. He needs help, not punishment.’ (informed) ‘That he has a cognitive disorder which meant that whilst he committed a crime, this was due to factors out of his control.’ (informed)
	ASD	‘The stress and possible autism.’ (uninformed) ‘Although he had reason to become annoyed he should not have hit anyone. However I am not fully convinced he does not have a condition that could have caused him to respond the way he did i.e. Could he have autism.’ (uninformed) ‘He displays lots of characteristics that you would expect from someone with a learning disability perhaps autism.’ (uninformed) ‘If it became apparent that he had a mental condition such as Asperger's syndrome then he wouldn't be wholly to blame for the incident.’ (uninformed)
	Mental health condition	‘Mr Edwards' actions were extreme. I felt he had lost control and may have either a mental health problem or a physical problem that would cause him to take extreme action. I thought his judgement was impaired.’ (uninformed) ‘Whilst reading the scenario it went through my head that he may have mental health issues and nobody picked up on that and jumped to conclusions and aggregated him. It should have been handled more sensitively at the station, before the police were called. He shouldn't have been charged again.’ (uninformed) ‘It's a difficult one really because he did hit a police officer but it should be taken into consideration that he may have mental health issues.’ (uninformed) ‘I think if he were to be assessed he would be found to have some sort of mental health issue.’ (uninformed)
Communication	Struggles to understand	‘Mr Edwards clearly struggles to fully understand questions and scenarios put to him, and then struggles to provide explanations.’ (informed) ‘He is unable to articulate his frustration and doesn't seem to completely understand the questions being asked of him.’ (informed) ‘He does seem to understand the questions but it takes him time to compute them.’ (informed) ‘He understands the questions but gets confused as to what is the right answer.’ (uninformed) ‘Irrational behaviour when questioned by police. Appears unable to answer simple questions.’ (uninformed) ‘I thought that Mr Edwards showed both when he was arrested and in court that he had some difficulty in understanding questions and giving precise answers. He was frustrated by the situation at the station and the fact that no one seemed to understand the difficulty he was experiencing.’ (uninformed)
	Articulation and expression	‘His responses aren't very articulate, suggesting to me weak cognitive functioning.’ (informed) ‘He appears to understand what happened and why, he just has issues expressing himself.’ (informed) ‘He does understand but isn't good at articulating his feelings and thoughts.’ (informed) ‘He is not able to form cohesive responses as he is upset, and the questioning is long. He knows the truth but can't articulate it.’ (uninformed) ‘Had reasoned responses but had difficulties articulating his feelings and understand the full question. Felt he had some level of understanding but acted with frustration and didn't know how to get others to understand.’ (uninformed) ‘It is evident that he has difficulty in articulating himself, it appears he becomes frustrated if rushed or not given adequate time to formulate his replay when asked questions. When the lawyer asks long questions, Mr Edwards becomes confused in his response.’ (uninformed)
Role of the ticket and police officers	Ticket and police officers at fault	‘The incident began because interlocutors were unable or unwilling to be patient with him as he tried to make himself understood. He lashed out when he was being restrained, and it is likely he didn't fully understand why that was happening. He probably felt he was acting in self‐defence. I would lay the blame at the feet of the arresting officers who were too quick to leap to the conclusion that he was being aggressive.’ (informed) ‘He didn't intend to hit or hurt anyone. it was not premeditated. A well‐trained police officer could have dealt with the situation better in the first please and not let it escalate.’ (informed) ‘I believe if he was able to communicate to the ticket officer his situation as a person without any cognitive diagnosis he would not have ended up in this situation. It is a shame the officer was not able to pick up that he had DLD.’ (informed) ‘The policeman's approach appeared overaggressive – it stemmed from an assumption of mentality normality, which isn't the case with this defendant.’ (informed) ‘He is clearly at fault for his own aggressive behaviour. However, it seems that the ticket officer may not have done a good job of understanding Mr Edwards problem, and also that the police may have failed to help diffuse it as well as they could have.’ (uninformed) ‘He shouldn't have physically hit the policeman, but I think the ticket officer should have tried harder to understand what the man was getting frustrated about.’ (uninformed)
